# Insights into enzymatic halogenation from computational studies

**DOI:** 10.3389/fchem.2014.00098

**Published:** 2014-11-11

**Authors:** Hans M. Senn

**Affiliations:** WestCHEM School of Chemistry, University of GlasgowGlasgow, UK

**Keywords:** enzymes, reaction mechanisms, DFT, QM/MM, ab initio methods, molecular modeling

## Abstract

The halogenases are a group of enzymes that have only come to the fore over the last 10 years thanks to the discovery and characterization of several novel representatives. They have revealed the fascinating variety of distinct chemical mechanisms that nature utilizes to activate halogens and introduce them into organic substrates. Computational studies using a range of approaches have already elucidated many details of the mechanisms of these enzymes, often in synergistic combination with experiment. This Review summarizes the main insights gained from these studies. It also seeks to identify open questions that are amenable to computational investigations. The studies discussed herein serve to illustrate some of the limitations of the current computational approaches and the challenges encountered in computational mechanistic enzymology.

## Chemistry of biohalogenation

Living organisms produce an astonishing array of halogenated compounds. Some 5000 halogen-containing natural products have been identified, which are biosynthesized by organisms from all domains of life, including bacteria, fungi, terrestrial plants, various marine organisms, and higher animals, including humans (Gribble, 1996, 1998, 2003, 2004, 2010). The halometabolites are not only highly diverse in biogenic origin, but also with respect to the halogen substitution patterns as well as chemical and structural complexity. They range from very simple molecules like CH_3_Cl to exceedingly structurally complex, highly functionalized natural products. Most halometabolites are biologically active, showing, e.g., antimicrobial, antifungal, or antibiotic activity. Their biological activity is usually critically dependent on the presence of the halogen(s). The most abundant halogen found in halometabolites is chlorine, followed by bromine, but there are also iodine- and even a few fluorine-containing compounds.

Until quite recently, little was known about the enzymes involved in the biosynthesis of natural organohalogens. The first halogenating enzymes to be discovered were the haem-dependent haloperoxidases in the 1960s; vanadate-dependent haloperoxidases followed in the 1980s. However, huge progress has been made over the last 10 years, with several entirely new families of halogenases being discovered and biochemically and structurally characterized. A number of reviews have summarized the findings from different perspectives (Anderson and Chapman, 2006; Vaillancourt et al., 2006; van Pée et al., 2006; Galonić Fujimori and Walsh, 2007; Blasiak and Drennan, 2008; Neumann et al., 2008; Butler and Sandy, 2009; Wagner et al., 2009; van Pée, 2012). The discoveries spurred an interest in biohalogenation and halogenases for a number of reasons. Firstly, some of the halometabolites are directly of interest for their biological activity. Secondly, the ability of many halogenases to install halogens very selectively in highly functionalized molecules, often at positions that would be difficult to access by conventional synthetic methods, could be exploited biocatalytically or biotechnologically (Deng et al., 2008b; Eustáquio et al., 2010; Runguphan et al., 2010; Herrera-Rodriguez et al., 2011a,b; van Pée, 2012; Payne et al., 2013; Smith et al., 2013). Thirdly, the recently discovered halogenases have revealed the fascinating variety of distinct chemical mechanisms that nature utilizes to activate halogens and introduce them into organic substrates. A detailed understanding of the mechanisms, the role played by the protein environment, and the determinants of selectivity are not only of fundamental interest for mechanistic enzymology, but will also inform the rational development of *in vitro* or *in vivo* uses of these unique biological catalysts.

Halogenases may be divided into three mechanistic classes according to the chemical nature of the active halogenating agent (Table 1). Solvated halide anions are the primary halogen source in all cases. They are activated in different ways for subsequent reaction with the organic substrate. The enzymes in each class can be grouped into families according to mechanism, co-factor, or type of substrate. Table 1 only covers halogenases whose structures are known experimentally as reliable protein structures are a prerequisite for the elucidation and modeling of enzymatic reaction mechanisms. However, other halogenases have been characterized genomically or biochemically, and it is clear that there exist other families or subfamilies not included here.

**Table 1:**
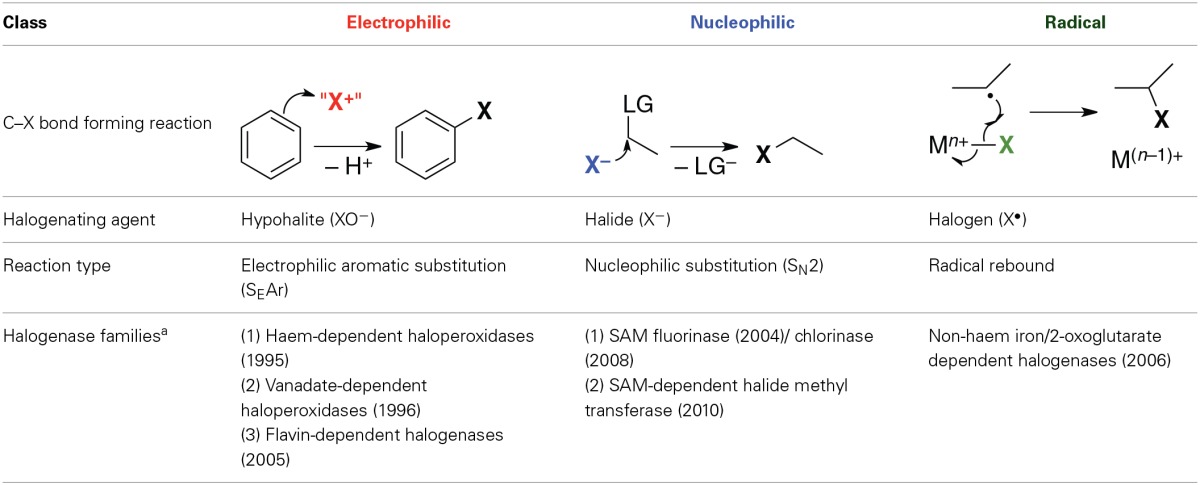
**Classes of halogenases according to chemical nature of the halogenating agent**.

*LG, leaving group; SAM, S-adenosylmethionine*.

*^a^ The year indicates the first structural characterization of an enzyme of the respective family*.

The purpose of this Review is to summarize computational studies on halogenases and the insight they have provided into the reaction mechanisms of these enzymes. The material is organized by mechanistic class and enzyme families as shown in Table 1. For each class, we will briefly outline the currently accepted, or generally proposed, mechanisms, before alighting on the computational studies and their main findings.

## Electrophilic halogenases

Electrophilic halogenases oxidize the halide anion to an electrophilic species “X^+^,” most likely hypohalous acid HOX (or hypohalite XO^−^, respectively), which then reacts with the organic substrate. These enzymes install the halogen at relatively electron-rich carbon centers, usually in alkenes or aromatic rings, *via* an electrophilic aromatic substitution reaction. The three structurally known families of electrophilic halogenases use different oxidants to effect the two-electron oxidation of X^−^ to “X^+^”: Haem-dependent and vanadate-dependent haloperoxidases require externally provided H_2_O_2_ and a metal co-factor while the flavohalogenases rely on their flavin co-factor, which *in vivo* is re-reduced for the next reaction cycle by a separate flavin-reducing enzyme.

### Haem-dependent haloperoxidases

**Figure 1:**
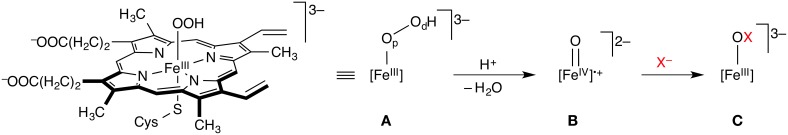
**Key steps of the mechanism of haem-dependent haloperoxidases**. The Fe^III^ hydroperoxido complex **A** (Compound 0) is oxidized to the Fe(IV) oxido species **B** (Compound I), which in turn oxidizes X^−^ to XO^−^. O_p_ and O_d_ in **A** designate the proximal and distal oxygen atoms, respectively.

The haem-dependent haloperoxidases (Fe-HPOs) feature a redox-active haem cofactor, akin to cytochrome P450 and related haem enyzmes (Figure 1). They depend on exogenous H_2_O_2_ to oxidize the haem-Fe center to the Fe(IV) oxido (“ferryl”) species **B** (also known as Compound I), which in turn oxidizes X^−^ (X = Cl, Br, I) to give HOX. Fe-HPOs lack a binding site for an organic substrate and have very poor substrate specificity and regio-/stereoselectivity. This suggests that HOX is released from the active site and subsequently reacts freely with whatever organic substrate it encounters that is susceptible to electrophilic attack. Depending on conditions and substrates present, Fe-HPOs promote a range of oxidation reactions other than halogenations.

The key steps involved in the oxidation of X^−^ to XO^−^ in Fe-HPOs essentially follow the chemistry of other haem enzymes, which has been extensively studied computationally (Shaik et al., 2005, 2009). Some of the same researchers have also turned their attention to Fe-HPOs, notably Shaik and co-workers. They used combined DFT/MM methods to study the formation of Compound I in chloroperoxidase (Chen et al., 2008), starting from the hydrogen peroxide adduct [Fe^III^]–(HOOH). The preferred pathway proceeds on the doublet (*S* = 1/2) surface via deprotonation of O_p_ to Compound 0 (**A** in Figure 1), followed by rate-determining heterolytic O–O cleavage with concomitant protonation of O_d_ and loss of water to form Compound I (**B**). This proton-catalyzed sequence, involving Glu183 as the general acid/base, was found to be preferred to direct O–O cleavage in [Fe^III^]–(HOOH). Also, reaction in the *S* = 1/2 (doublet) electronic state is favored over *S* = 3/2 (quartet) or *S* = 5/2 (sextet) reactivity. In contrast to other P450-type enzymes, Compound I of chloroperoxidase is experimentally accessible and spectroscopically well characterized (Jung et al., 2011). Its ground state is a doublet, in agreement with the calculations. Using combined CASPT2/MM (Chen et al., 2010b), it was shown that DFT (specifically, B3LYP) by and large provides an adequate description of the intricate electronic structure and energetics of these systems. The reaction from Compound 0 to Compound I is an interesting case of a proton-coupled electron transfer (PCET). The early stage of the O–O cleavage is homolytic, but the proton transfer to O_d_ triggers the transfer of an electron from the [Fe]–O_p_ unit to the incipient water molecule, which makes the overall process heterolytic.

A follow-up study from the same group (Lai et al., 2009a) reported further details about the effects of the substrate, protonation state of active-site residues, and type of ligand *trans* to the oxygen ligands on the formation of Compound I in chloroperoxidase. Another contribution from the Shaik group (Lai et al., 2009b) used again DFT/MM methods to compute Mössbauer parameters of different isomers and protonation states of Compound II, {[Fe^IV^]=O}^3−^, of chloroperoxidase (Compound II is derived from Compound I by one-electron reduction). The calculations allowed the assignment of three different Compound II species in the experimental spectrum.

### Vanadate-dependent haloperoxidases

**Figure 2:**
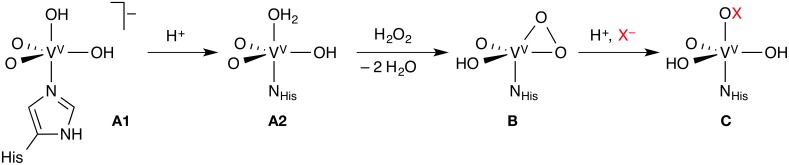
**Key steps of the mechanism of vanadate-dependent haloperoxidases**. Each complex may exist in different protonation states, only one of which is shown; the exact order of the reaction steps leading from **A1** to **B** is uncertain.

Like the Fe-HPOs, the vanadate-dependent haloperoxidases (V-HPOs) use exogenous H_2_O_2_, but activate it as a vanadium(V) peroxido complex, which then oxidizes X^−^ (X = Cl, Br, I) to HOX (Figure 2). The vanadate co-factor acts as a Lewis base, the metal center remaining as V(V) throughout the catalytic cycle, unlike the redox-active iron center of the Fe-HPOs. There are indications, at least in some V-HPOs, that HOX does not escape from the enzyme completely, but remains associated with the enzyme in some way. This may explain why V-HPOs are generally more selective and less promiscuous than Fe-HPOs. V-HPOs have been shown to be involved in the biosynthesis of halometabolites in marine organisms.

The majority of the computational studies of vanadate-dependent haloperoxidases have focused on elucidating the protonation states of the different intermediates of the co-factor. Because the vanadate is surrounded by several titratable, hydrogen-bonding residues, studies of the isolated co-factor, or even small models including a few residues, are of limited value. We will therefore only discuss results from combined DFT/MM studies that consider the entire protein, or at least large parts of it. It should be noted, however, that the assignment of protonation states remains a difficult problem even at this rather detailed level of modeling. Slight differences in the computational method (e.g., different exchange-correlation functionals in DFT) or in the model setup (in particular, differences in protonation states in the MM part of the model) can change the outcome. It is often impossible to resolve such problems without recourse to experimental data.

A number of studies looked at the resting state (**A** in Figure 2) of vanadium chloroperoxidase (V-CPO), which is well characterized by X-ray crystallography, UV/Vis, and solid-state NMR spectroscopy. The identity of **A** could only be determined with reasonable certainty through the combination of computational and experimental results. A series of papers from the Bühl group (Waller et al., 2007, 2008; Geethalakshmi et al., 2009) concluded that computed ^51^V NMR isotropic chemical shifts, even in combination with structural data, were not sufficient to assign the protonation states with certainty. Only by calculating additional NMR parameters (chemical shift anisotropies and nuclear quadrupole couplings) can one resolve the question. The consensus from several studies (Kravitz et al., 2005; Raugei and Carloni, 2006; Waller et al., 2007; Zhang and Gascón, 2008) is that the resting state of V-CPO is most likely the doubly protonated monoanion **A1**.

The reaction proceeds by protonation from **A1** to **A2**, followed by attack of H_2_O_2_ and loss of two water molecules to yield the η^2^-peroxido complex **B** (Kravitz et al., 2005; Raugei and Carloni, 2006). However, the order of these steps is not entirely clear. The protonation state of complex **B**, whose structure is known, is again ambiguous; the neutral, singly protonated form shown in Figure 2 appears to be most likely (Geethalakshmi et al., 2009). While the studies on V-CPO discussed so far were devoted to the identification and characterization of particular intermediates, one study modeled the complete catalytic cycle of V-CPO (Raugei and Carloni, 2006), even though with bromide as the halide.

In a similar vein to their work on V-CPO, the Bühl group also investigated vanadium bromoperoxidase (V-BPO). The ^51^V NMR isotropic chemical shift of the resting state was again found to be insensitive to the protonation state, requiring a combination of additional NMR parameters for a clear signature (Waller et al., 2008). The substantial shielding of the ^51^V signal in V-BPO compared to V-CPO could not be reproduced computationally. Moreover, when going from solution to the solid state, the ^51^V resonance of V-BPO is exceptionally deshielded, in contrast to V-CPO, where there is no significant change. No satisfactory explanation of this effect could be provided. For the peroxido vanadate intermediate of V-BPO, the same form as in V-CPO was identified (Geethalakshmi et al., 2009).

Pacios and Gálvez (2010) built a homology model of vanadium iodoperoxidase (V-IOP) and investigated the selectivity for iodide over bromide and chloride by means of electrostatic potentials calculated on molecular surfaces. They also studied the isolated co-factor by DFT calculations. They propose that the mutation of an active-site Ser (in V-CPO and V-BPO) to Ala in V-IOP lowers the electrostatic potential in the halide binding site, favoring the less electronegative iodide over bromide or chloride. V-IPO also has a more open, easily accessible active site.

### Flavin-dependent halogenases

**Figure 3:**
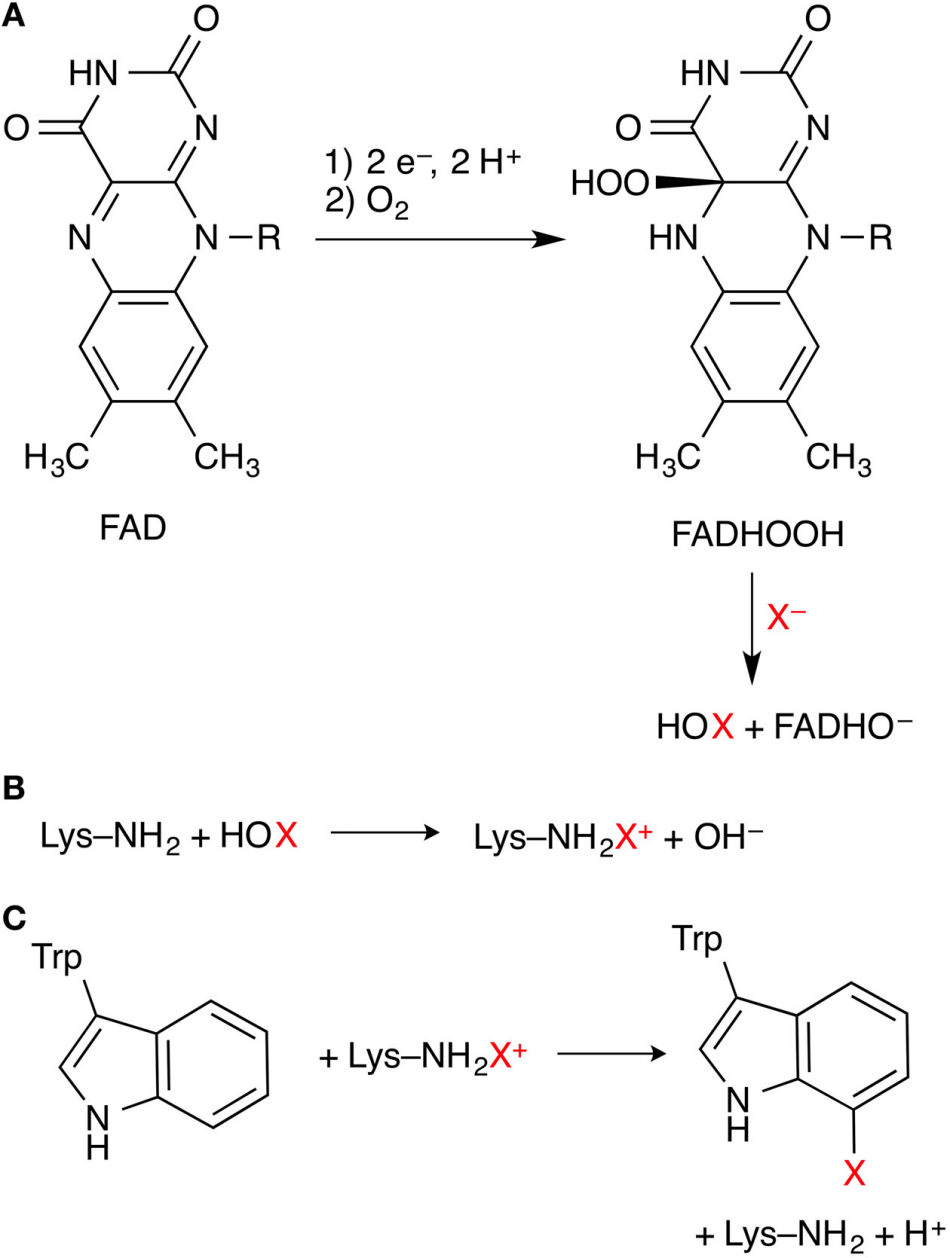
**Key steps of the mechanism of flavin-dependent tryptophan 7-halogenases. (A)** Formation of FADHOOH, which oxidizes X^−^ to HOX. **(B)** Proposed formation of a lysine *N*-haloamine intermediate. **(C)** Electrophilic aromatic substitution of the substrate tryptophan.

Flavin-dependent halogenases (FDHs) use a flavin co-factor (FAD, flavin adenine dinucleotide) to activate dioxygen in the form of a hydroperoxyflavin (FADHOOH) intermediate. The steps leading to this intermediate are well-known from other flavoenzymes, in particular FAD-dependent monooxygenases. FADHOOH acts as a source of “OH^+^” (Figure 3A) and is able to hydroxylate a halide anion X^−^ (X = Cl, Br) that is bound in close proximity to the co-factor, affording hypohalous acid HOX. In contrast to the haloperoxidases, the formed HOX is not released, but reacts in a second active site, some 10 Å away from the flavin/X^−^ binding site, where the organic substrate is bound. In the currently favored mechanism, HOX initially reacts with the side-chain amino group of a conserved lysine residue in the lining of the channel that connects the two binding sites (Figure 3B). The resulting *N*-haloamine (–NH_2_X^+^ or –NHX) intermediate then serves as the actual electrophilic halogenation agent that transfers “X^+^” onto the aromatic substrate (Figure 3C).

FDHs are highly substrate- and regio-/stereoselective. Five enzymes of this family have so far been characterized structurally. They represent two variants (Buedenbender et al., 2009; Podzelinska et al., 2010). Variant A acts on free “small-molecule” substrates: PrnA (Dong et al., 2005) and RebH (Yeh et al., 2007) halogenate free tryptophan regioselectively at position 7 of the indole ring while the closely related PyrH (Zhu et al., 2009) is a tryptophan 5-halogenase. CmlS halogenates an activated carbon center adjacent to a carbonyl group, however, the exact form of the natural substrate is unclear. CmlS (Podzelinska et al., 2010) is different in that the FAD co-factor is covalently bound to the protein. Variant B FDHs only accept amino-acid substrates tethered to a carrier protein (similarly to the non-haem iron/2-OG dependent halogenases, see Section Radical halogenases). The only structurally characterized representative of this kind is the tyrosine 2-halogenase CndH (Buedenbender et al., 2009), in which FAD is also covalently bound to the protein.

FDHs have raised a number of interesting questions amenable to computational investigations: (i) Identity of the halogenating agent: Does the reaction indeed involve an *N*-haloamine intermediate, or is HOX activated and controlled by hydrogen-bonding to the lysine side-chain amine, without forming an *N*-haloamine? (ii) Protonation states, involvement of general acid/base residues: The proposed mechanism includes several species that can exist in different protonation states (FADHO[H], [H]OX, Lys–NH_2_[H]^+^, Lys–NH[H]X^+^); also, the formation of “X^+^” from HOX generates OH^−^, while the electrophilic aromatic substitution step releases H^+^. In the case of acyl halogenation in CmlS, the currently proposed mechanism involves the formation of a substrate enolate, implying deprotonation of the activated carbon. What are the actual protonation states; what proton transfer steps are involved in the catalytic cycle; which protein residues are acting as proton donors/acceptors? (iii) Regioselectivity: In the tryptophan halogenases, what factors control whether the substrate is 5- or 7-halogenated; how does the enzyme overcome the inherent chemical selectivity preference of the substrate? This is of particular relevance as other, not yet structurally characterized Trp halogenases are known that are 6- and 2-selective, respectively. However, despite the availability of several high-resolution structures of FDHs representing a range of substrate- and regioselectivities and plenty of biochemical data, we are not aware of any published computational studies on flavin-dependent halogenases.

## Nucleophilic halogenases

**Figure 4:**
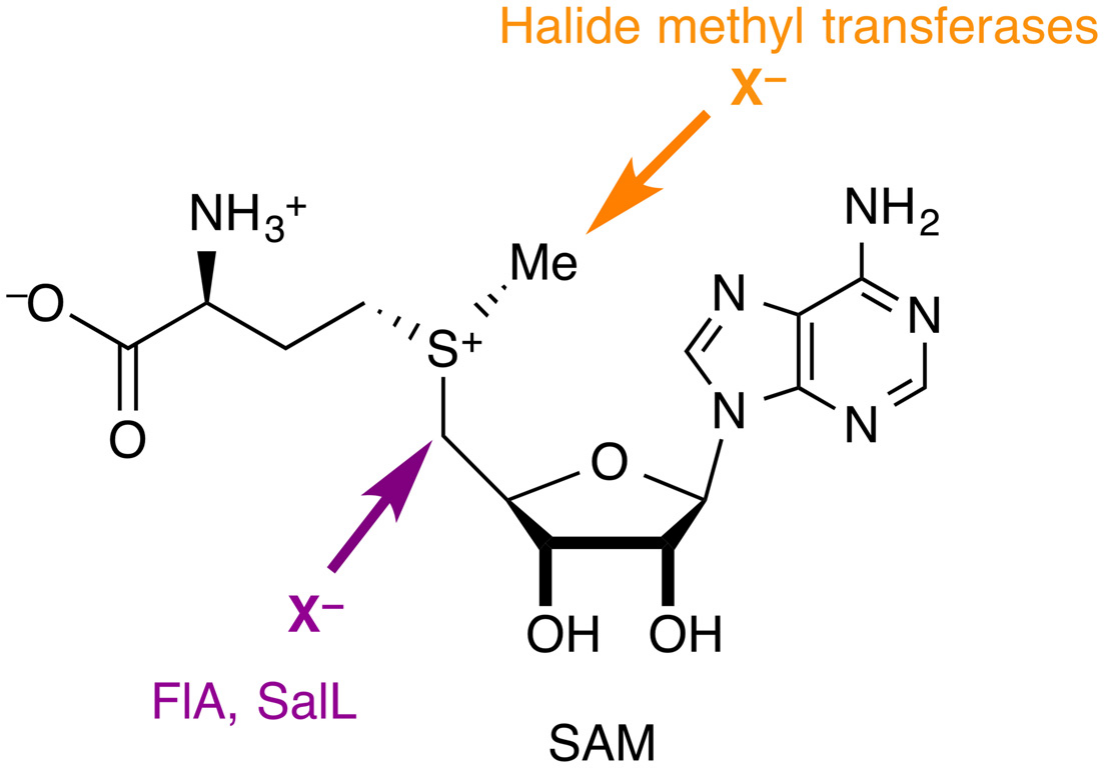
**Nucleophilic halogenases catalyze the attack of halide anions as nucleophiles on SAM**. The fluorinase FlA and the chlorinase SalL produce halogenated adenosines, whereas halide methyl transferases produce halomethanes.

Although halide anions are not normally considered viable nucleophiles, especially in aqueous solution, there are halogenases that use (partially) desolvated X^−^ anions (X = F, Cl, Br, I) as nucleophiles in substitution reactions at carbon. In all the cases identified so far, the substrate is *S*-adenosyl-l-methionine (SAM), whose carbon centers adjacent to the sulfonium are strongly electrophilic by virtue of the positive charge on sulfur and the favorable leaving group characteristics of the thioether (Figure 4). Two families of nucleophilic halogenases have been characterized structurally, which we discuss in turn below.

### SAM fluorinase and SAM chlorinase

The fluorinase FlA (or 5′-fluoro-5′-deoxyadenosine synthase, FDAS), the only native fluorinating enzyme identified to date (Dong et al., 2004), accepts F^−^ and Cl^−^ as halide substrates. The structurally very similar chlorinase SalL accepts Cl^−^, Br^−^, and I^−^, but not F^−^. The products of both enzymes are the corresponding halogenated adenosine derivatives and l-methionine. Notably, SAM is a substrate in these reactions, rather than a co-factor, unlike in the SAM-dependent halide methyl transferases discussed in Section SAM-dependent halide methyl transferases.

The C–F bond-forming step in the fluorinase was studied in detail by combined DFT/MM calculations (Senn et al., 2005). For comparison, the intrinsic reactivity of SAM + F^−^ was investigated by DFT calculations with a polarizable continuum model for water. As no experimental structure of the enzyme with bound fluoride is available, the anion binding site was explored by classical MD simulations and quantum mechanics/molecular mechanics (QM/MM) optimizations, which identified the crucial role of hydrogen-bonding in stabilizing the fluoride anion in the active site. The C–F bond is formed *via* an S_N_2-type nucleophilic attack of fluoride on SAM, the methionine thioether being the leaving group. Compared to (polarizable continuum) water, the potential-energy barrier of this step is lowered by 40–50 kJ mol^−1^ in the enzyme. Thanks to enzyme catalysis, the actual C–F bond formation is thus a facile step and not rate-limiting for the overall fluorination process. The free-energy barrier, calculated by QM/MM free-energy perturbation (FEP) (Senn et al., 2009), is only a few kJ mol^−1^ lower than the potential-energy barrier. However, there will clearly be substantial entropic and finite-temperature effects on the steps preceding and following the bond-forming event; in particular, binding and desolvation of fluoride and product release. As these processes are likely to be coupled to large-scale conformational changes (e.g., tertiary-structure rearrangements), they are difficult to model computationally. Experimentally, the reaction is very slow and inefficient: *k*_cat_ = 0.07 min^−1^, *k*_cat_/*K*_m_ = 10^4^ L mol^−1^ min^−1^ (Zhu et al., 2007). The rate-determining step is unknown, but it clearly is not C–F bond formation.

FlA was shown experimentally to be able to act also as a chlorinase (Deng et al., 2006), however, with the equilibrium of the chlorination reaction being shifted to the reactant side. This is consistent with the calculated (Senn et al., 2009) endergonic reaction energy and a higher barrier for chlorination compared to fluorination. Computationally, carbon–halogen bond formation/cleavage should also be feasible with bromide and iodide, with the equilibrium shifted even further toward reactants (Senn et al., 2009); however, no reactivity was observed experimentally with either of the heavier halides. A possible explanation is that halide selectivity may not be determined in the C–X bond-forming step, which was the focus of the computational studies, but in a preceding step, e.g., halide binding.

A small number of sequence and structural homologs of the fluorinase FlA are known. The chlorinase SalL produces 5′-chloro-5′-deoxyadenosine from SAM and chloride, using an active-site setup highly similar to the fluorinase's (Eustáquio et al., 2008b). SalL does not accept fluoride as a substrate, but is similarly active for chlorination, bromination, and iodination. For the Tyr70Thr mutant of SalL, which has been structurally characterized with SAM and chloride ion bound in the active site, the chlorination barrier was calculated by DFT/MM (Healy, 2011). In addition to confirming the S_N_2-type attack of the halide, that study focused on the role active-site water might play. In contrast to the wild-type, the Tyr70Thr mutant contains a water molecule in the active site, which hydrogen-bonds to the chloride ion at the expense of the interaction between chloride and a back-bone amide NH. The “microsolvation” of the chloride by one water molecule in the active site, which will reduce its nucleophilicity, may account for the significantly lower activity of the mutant compared to the wild-type. Moreover, the presence of water may have further ramifications (Healy, 2011). By exposing the back-bone NH that was previously involved in binding the halide, the water may indirectly disturb the protein's local secondary structure. This notion was supported by classical MD simulations on the Ser158Gly mutant of FlA (Healy, 2011). In a simulation of the apo form (obtained by removing bound inhibitor and chloride from the crystal structure), a water molecule initially occupied the halide binding site. However, this interaction was subsequently lost, exposing a back-bone NH and thus causing a local distortion of the secondary structure.

Genome sequencing has revealed that both FlA and SalL belong to the DUF62 superfamily of proteins, which contains about 200 members. Five other DUF62 proteins have been structurally characterized and/or assayed for their function (Deng and O'Hagan, 2008; Deng et al., 2008a; Eustáquio et al., 2008a). Despite very close structural similarity to FlA and SalL, these enzymes show no halogenating activity, but catalyze the hydrolysis of SAM into adenosine and methionine. None of them has so far been the subject of computational mechanistic studies, which could reveal the origin of the different substrate- and chemoselectivities.

Chemically related to the fluorinase is the fluoroacetate dehalogenase, which catalyzes the hydrolytic defluorination of fluoroacetate into hydroxyacetate (glycolate) and fluoride. Its mechanism has been investigated in detail by QM/MM calculations (Kamachi et al., 2009). The reaction sequence follows the paradigm of the related haloalkane dehalogenases (which, however, are unable to process fluorinated substrates): The fluoride is displaced by a side-chain carboxylate in an S_N_2-type reaction, yielding an ester intermediate, which is subsequently hydrolyzed *via* a tetrahedral intermediate. The latter step was found to be rate-determining, while halide displacement is facile.

### SAM-dependent halide methyl transferases

Harnessing the power of SAM as a versatile biological methylating agent, SAM-dependent halide methyl transferases produce halomethanes, CH_3_X (X = Cl, Br, I). Although biochemically characterized in the 1990s and quite possibly the most prevalent kind of halogenases, the first structure of a member of this family only appeared in 2010 (Schmidberger et al., 2010). We are unaware of any computational work on these enzymes.

## Radical halogenases

### General mechanism of non-haem iron/2-oxoglutarate dependent halogenases

**Figure 5:**
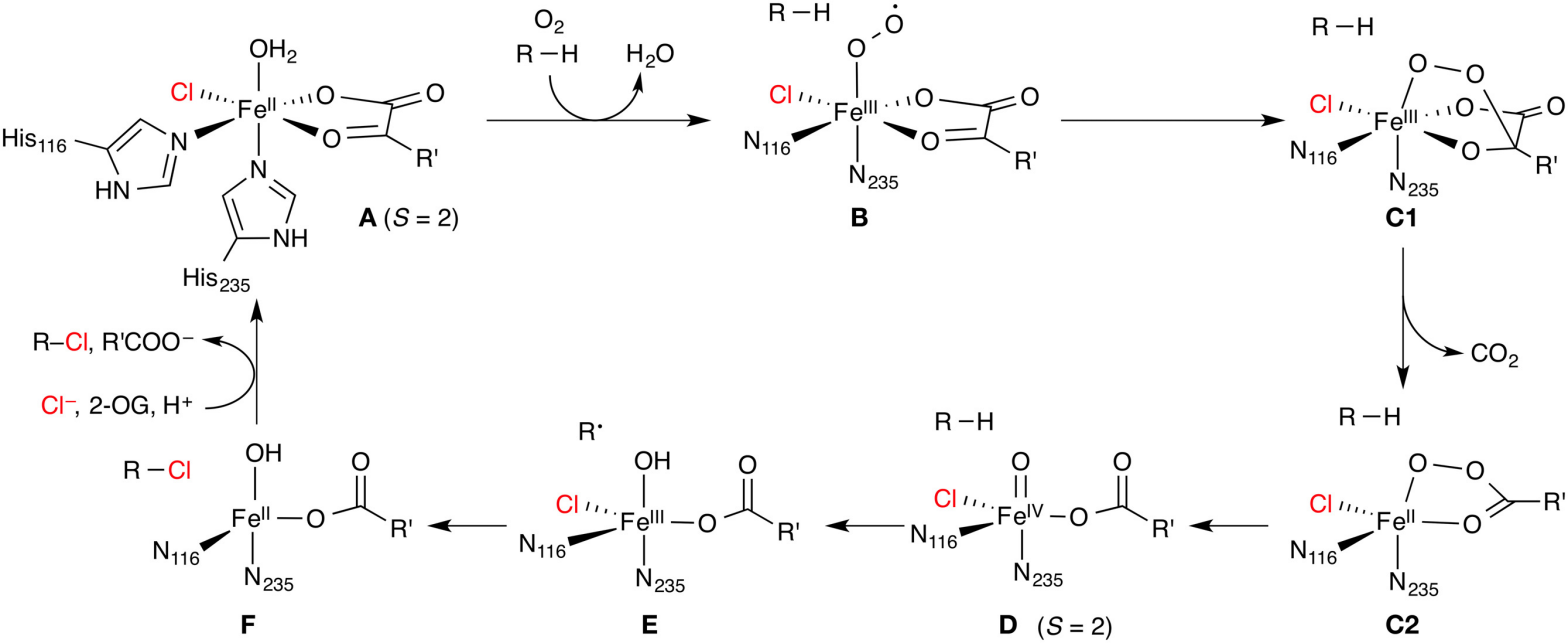
**Proposed catalytic cycle of non-haem iron/2-oxoglutarate (2-OG) dependent halogenases**. R is an aliphatic carbon center of the substrate; R' = (CH_2_)_2_COO^−^. The numbering of His residues is taken from SyrB2.

The enzymes in this class are non-haem iron/2-oxoglutarate (2-OG, “α-ketoglutarate”) dependent and rely on the mononuclear iron complex to install a halogen (Cl or Br) at non-activated carbon centers *via* a radical mechanism. 2-OG and O_2_ are required as co-substrates. The proposed catalytic cycle (Figure 5) is based on the well-studied mechanism of the non-haem iron/2-OG dependent hydroxylases. However, in the hydroxylases (whose prototypical representative is the taurine hydroxylase TauD), the iron is coordinated by a “facial triad” of two histidine side chains and a side-chain carboxylate (Asp or Glu) whereas the halogenases have two histidine and a halide ligand. Starting from the resting state **A** with a water-coordinated Fe(II) center, the water is displaced by substrate binding and O_2_ is coordinated. The O_2_-bound complex is most commonly formulated as an Fe(III) superoxido complex **B** (with *S* = 2). Further reduction of the O_2_ moiety and attack of the distal oxygen on the keto carbon of 2-OG leads to a peroxy structure **C**. Oxidative decarboxylation affords the high-spin (*S* = 2) Fe(IV) oxido (“ferryl”) species **D**, coordinated to succinate. **D** is powerful enough an oxidant to abstract a hydrogen atom from an aliphatic carbon center, R–H. The substrate radical R^•^ thus generated recombines with the iron-bound halogen in a rebound step to form the halogenated product R–X.

**Figure 6:**
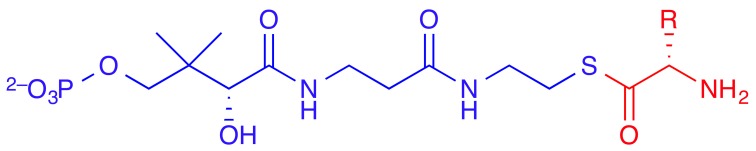
**The (amino-acid) substrate (shown in red) of non-haem iron/2-oxoglutarate dependent halogenases is linked as a thioester to a phosphopantetheine (PPant) tether (in blue) and presented to the enzyme by a carrier protein**.

The native substrates of most non-haem iron/2-OG-dependent halogenases (NHFeHs) are α-amino acids, which are halogenated at aliphatic side-chain positions, usually a terminal carbon. Three enzymes of this family have been characterized structurally: SyrB2 (Blasiak et al., 2006), which chlorinates the terminal C^γ^ of l-threonine (l-Thr); its close homolog CytC3 (Wong et al., 2009), which double-chlorinates the terminal C^γ^ of (2*S*)-2-aminobutanoate (l-Aba); and the halogenation domain of CurA (Khare et al., 2010). The latter installs chlorine at C2 of (3*S*)-3-hydroxy-3-methylglutarate and thus works on a different type of substrate. In all cases known to date, the free substrates are not recognized by the enzyme. Rather, the substrate needs to be linked as a thioester to a phosphopantetheine (PPant) tether (Figure 6) and presented to the halogenase by a separate carrier protein. There is no experimental structure of a substrate-bound NHFeH.

A key question arising from the mechanism shown in Figure 5 is why the substrate radical combines preferentially with the chlorido, rather than the hydroxido, ligand (**E** → **F**). It is known experimentally for SyrB2 that certain non-natural substrates are indeed hydroxylated, which demonstrates that these halogenases are in principle competent to catalyze either reaction (Matthews et al., 2009b). An important piece of evidence from Mössbauer spectroscopy is that two forms of **D** exist in equilibrium (Galonić et al., 2007; Matthews et al., 2009a). The intermediate **D** is relatively stable and has been experimentally characterized in NHFeHs and closely related NHFe hydroxylases. **D** can therefore serve as a “stepping stone” in mechanistic studies, and it is convenient to divide the discussion of the mechanism into two sections: (1) Formation of the Fe(IV) oxido intermediate (**A** → **D**, Section Formation of the Fe(IV) oxido intermediate); and (2) hydrogen abstraction and radical rebound (**D** → **F**, Section Hydrogen abstraction and radical rebound). However, before presenting mechanistic results, we turn to the problem of substrate placement in the NHFeH active site.

### Substrate placement in NHFeHs

**Figure 7:**
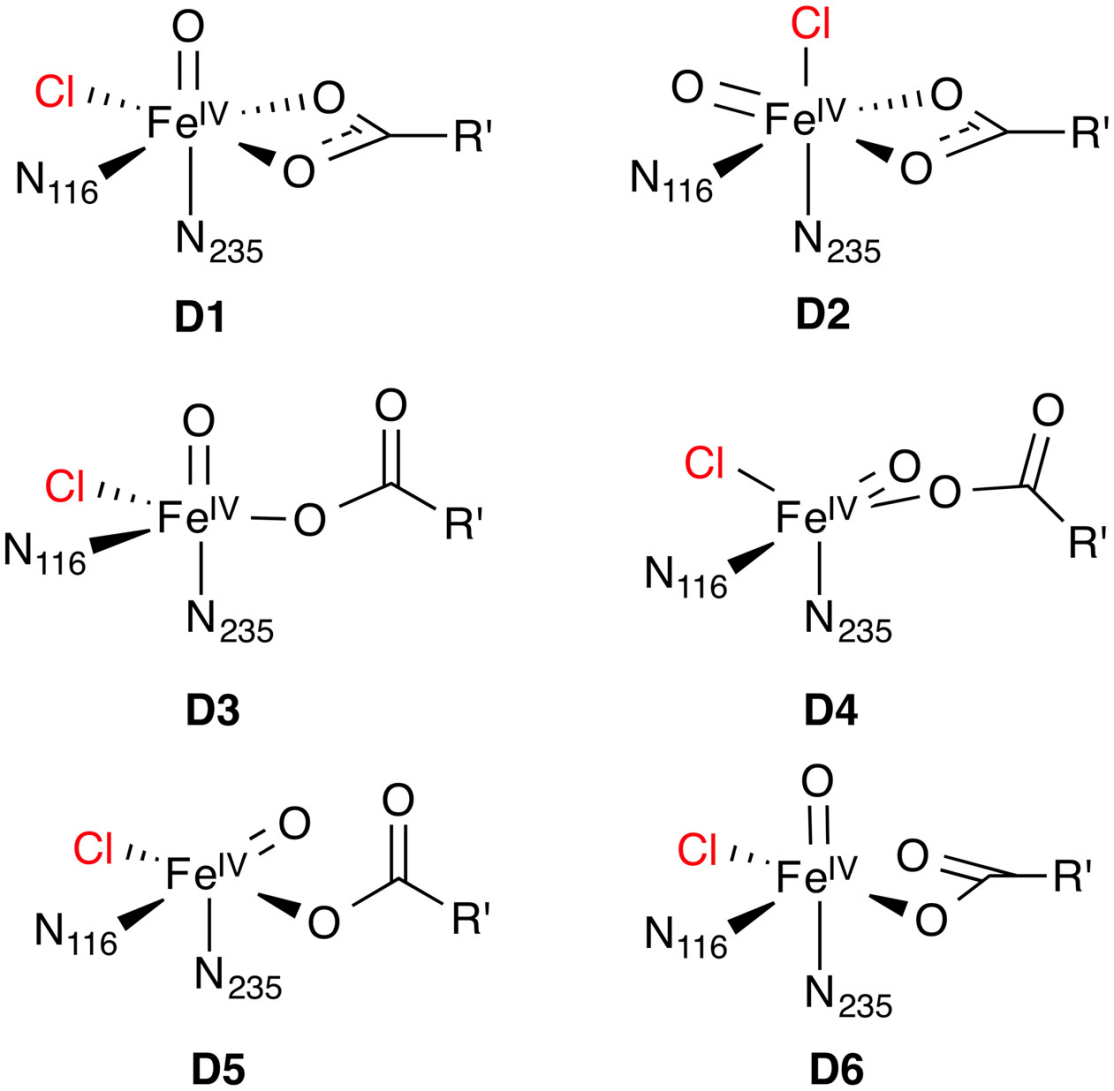
**Possible isomeric structures of the ^5^[Fe^IV^ =O] species D; R' = (CH_2_)_2_COO^−^**.

As no experimental structure of a substrate-bound NHFeH is available, a few studies applied molecular-docking procedures to place the substrate in the active site. Borowski et al. (2010) used AutoDock 4 to position l-Thr linked to a truncated PPant tether into the crystal structure of holo-SyrB2, which contained complex **A** without the water ligand. They selected a pose where the reactive C^γ^ methyl group of l-Thr was positioned in proximity to the iron center and which was low in energy (in the lowest-energy pose, C^γ^ was deemed too far away). Based on the substrate-bound structure, they built a cluster model to study the reaction steps **D** → **F** (see Section Hydrogen abstraction and radical rebound) after manually converting **A** to **D1** (see Figure 7). Solomon and co-workers (Wong et al., 2013) subsequently used Borowski's docked structure as a starting point in their studies of the SyrB2 mechanism.

In an extensive modeling exercise combined with site-directed mutagenesis experiments (Fullone et al., 2012), homology models were constructed of the threonine NHFeH Thr3 and the PPant-binding T domain of the carrier protein SyrB1; the complexes between SyrB2 or Thr3 and SyrB1-T were then modeled; and the Thr-loaded PPant arm was docked into the crystal structure of SyrB2, again using AutoDock 4. The protein–substrate and protein–protein contacts identified in the modeling studies were validated by site-directed mutagenesis. The enzyme–substrate complex largely agreed with Borowski et al. (2010).

Kulik and Drennan (2013) used Glide to dock Thr-PPant into SyrB2, then cut large (541 atoms) cluster models from the docked complexes, and optimized them at DFT level. They selected the lowest-energy one to build a smaller cluster model, which was then used to study the effect of substrate position on the hydrogen abstraction step (see Section Hydrogen abstraction and radical rebound).

### Formation of the Fe(IV) oxido intermediate

#### O_2_ activation and decarboxylation

The details of the reduction of O_2_ and generation of the Fe(IV) oxido intermediate in NHFe/2-OG dependent enzymes have found considerable attention from both the experimental and computational side. It is generally assumed that this part of the catalytic cycle follows the same, or a very similar, mechanism in NHFe hydroxylases and NHFeHs. An excellent overview of the computational studies on the formation of the Fe(IV) oxido intermediate in NHFe hydroxylases has very recently been prepared by Siegbahn and co-workers (Blomberg et al., 2014). In brief, there are two main types of mechanisms, which will be summarized below. In all cases, the starting point is **A** in the *S* = 2 state (or its 2-His/1-carboxylate congener, respectively) after displacement of water, for which we introduce the shorthand ^5^[Fe^II^]. Triplet dioxygen binds to the vacant coordination site of ^5^[Fe^II^] previously occupied by the water ligand, and through a sequence of steps, the Fe(IV) oxido complex in the quintet state, ^5^[Fe^IV^ =O] (**D**), is generated. The reaction **A** → **D** is strongly thermodynamically favored, by about −250 to −300 kJ mol^−1^.

The first type of mechanism (Mechanisms 1, 2, and 3 in the numbering of Blomberg et al., 2014) has been derived from several B3LYP studies done over a period of some 10 years in different groups, notably Siegbahn's (Bassan et al., 2004; Borowski et al., 2004a,b) and Neese's (Ye et al., 2012). The calculations used small models of the NHFe hydroxylase active site, with imidazole ligands modeling His, acetate modeling Asp/Glu, a (truncated) 2-oxocarboxylate, and sometimes one or two other side-chain models. In this mechanism, there exists a stable O_2_-bound complex of type **B** with three close-lying spin states: ^3^[Fe^II^(O_2_)], ^5^[Fe^III^(O^•−^_2_)], and ^7^[Fe^II^(O_2_) ↔ Fe^III^(O^•−^_2_)]. Only the quintet and septet are reactive; the reaction cannot progress from the triplet. On the quintet and septet surfaces, the reaction proceeds by nucleophilic attack of the distal oxygen on the keto carbon to a structure of type **C1**, which may be either a transition state or an intermediate, depending on the details of the method and the model. On the septet surface, decarboxylation and O–O bond cleavage occur concomitantly, leading directly to an “oxyl” form of **D**, ^7^[Fe^III^–O^•^], which (*via* spin–orbit coupling) rapidly relaxes to ^5^[Fe^IV^ =O] (**D**). In the quintet state, decarboxylation affords an intermediate with chelating peroxysuccinate, ^5^[Fe^II^(OOsucc)] (**C2**). This is separated from ^5^[Fe^IV^ =O] by small barriers for O–O cleavage, which occurs in two consecutive one-electron steps. The first step in this mechanism (attack of the distal oxygen with concomitant decarboxylation) is rate-determining, with a barrier of about 45–65 kJ mol^−1^. The quintet and septet pathways are equally competitive.

The sequence of steps outlined above may be considered a kind of “B3LYP consensus” mechanism. However, it should be noted that other studies (Topol et al., 2006; de Visser, 2007) used essentially the same methods and models but found a higher barrier for O–O cleavage, which may even become rate-determining. The reason for these discrepancies is not currently apparent. However, all B3LYP studies taken together have yielded a broadly consistent description of the reaction sequence: ^5^**A** → ^5,7^**B** → ^5,7^**C1** (→^5^**C2**) → ^5^**D**.

The “B3LYP consensus” mechanism is further supported by results from high-level *ab initio* calculations (Ye et al., 2012) on a minimal model (His replaced by NH_3_, Asp by OH^−^, 2-OG by 2-oxopropanoate; the model was validated at the B3LYP level). Dioxygen binding and the electronic structure of the O_2_-bound species **B** were studied with CASSCF and NEVPT2 multireference methods. (NEVPT2 stands for *n*-electron valence state perturbation theory to 2nd order and is essentially a multireference version of MP2. It may be considered a more computationally robust variant of CASPT2.) Moreover, the spin-splitting energies for **B** as well as the energies of all stationary points along the quintet and septet reaction paths were calculated with CCSD(T). As CCSD(T) is a single-reference method, its suitability was validated by calculating the spin-splitting energies of **D** at the multireference level using CASSCF and SORCI (SORCI stands for spectroscopy-oriented configuration interaction and is a variant of MRCI). As is to be expected, there are quantitative differences between the methods. For instance, the *S* = 1, 2, and 3 states of **B** were found (Ye et al., 2012) to lie within 6 kJ mol^−1^(B3LYP), 15 kJ mol^−1^(B3LYP), and 26 kJ mol^−1^[CCSD(T)], but all methods agree on the triplet ground state. Similarly, the rate-determining barrier **B** → **C1^‡^** is nearly twice as high at the B3LYP level (41 kJ mol^−1^) compared to CCSD(T) (23 kJ mol^−1^). However, the high-level calculation essentially confirm the B3LYP results.

The uncertainties about the exact mechanism of the conversion **A** → **D** are exacerbated by the lack of experimentally characterized O_2_-bound intermediates for NHFe/2-OG hydroxylases or halogenases. Instead, more stable NO complexes have been studied spectroscopically, which in terms of their electronic structure can be considered one-electron-deficient analogs of the O_2_ complexes. It has even been possible to characterize a one-electron reduced NO-bound intermediate (Ye et al., 2010), which is isoelectronic to an O_2_ complex. Calculations of various spectroscopic properties of NO complexes have served to validate computational methods against experiment. In particular, B3LYP was found accurately to reproduce Mössbauer parameters (Ye et al., 2010). With the assumption that the spectroscopic properties are sensitive to the electronic structure, this lends further support that B3LYP is correctly able to describe also O_2_-bound complexes and their reactivity.

The second type of mechanism proposed for the reaction **A** → **D** (Mechanism 4 in Blomberg et al., 2014) has been derived by Solomon and co-workers (Diebold et al., 2011) based on calculations with an *ad-hoc* hybrid DFT method using BP86 + 10% Hartree–Fock (HF) exchange (for comparison, B3LYP contains 20% HF exchange). They “spectroscopically calibrated” this particular method against UV/Vis absorption and EPR data of NO-bound biomimetic complexes (Schenk et al., 2004) and a hydroxylase (Diebold et al., 2011). They specifically discarded B3LYP as it yielded a qualitatively different electronic structure incompatible with the spectral data. With BP86 + 10% HF exchange, addition of O_2_ to **A** affords superoxido complexes of type **B** on the quintet and septet surfaces, similar to those obtained with B3LYP. However, for the *S* = 1 ground state, the reaction proceeds directly to an Fe(IV) peroxy-bridged structure of type **C1**. Through a triplet–quintet spin-crossing point, which is structurally still of type **C1** and about 45 kJ mol^−1^ higher in energy, a peroxyacid intermediate of type **C2** is obtained. The decay of the latter is practically barrier-free, leading to **D**. So the reaction sequence can be summarized as: ^5^**A** → ^3^**C1** → ^5^**C2** → ^5^**D**.

While the studies discussed so far used the 2-His/1-carboxylate facial triad typical of NHFe/2-OG hydroxylases, two studies were devoted specifically to the halogenases with their 2-His/X ligand sphere. Kulik et al. (2009) investigated the entire pathway **A** → **F** on a small active-site model including only the first-shell ligands, comparable to the models in the studies above. They employed a DFT method with a self-consistent Hubbard-type *U* correction (specifically, PBE+*U*) and a plane-wave basis set. This kind of approach is common in solid-state calculations, but rarely seen in molecular applications. In essence, the occupation-dependent *U* term compensates for some of the deficiencies of pure exchange-correlation functionals due to the self-interaction error. It significantly improves the description of electronic and structural features of transition-metal complexes compared to pure functionals (Kulik et al., 2006; Kulik and Marzari, 2008) while maintaining their computational expediency. At the PBE+*U* level (Kulik et al., 2009), the O_2_ adduct has a quintet ground state (with a close-lying triplet), and the reaction proceeds on the quintet surface without barrier from the addition of O_2_ directly to **D**, with concomitant decarboxylation and O–O bond cleavage: ^5^**A** → ^5^**D**.

The second study using a halogenase model stems again from the Solomon group (Wong et al., 2013), who used pure BP86 to study the oxygen activation and decarboxylation steps. Although building directly on their earlier hydroxylase work discussed above (Diebold et al., 2011), they did not further justify why they chose BP86 over BP86 + 10% HF in this case. Their model included some second-sphere active-site residues that are involved in hydrogen bonding to the substrate or the first-sphere complex. They also determined the position of the substrate from a docking procedure. In addition to the natural substrate l-threonine, they investigated two non-natural ones. Notwithstanding the difference in method, they found the same reaction pathway as in their hydroxylase study: Addition of O_2_ affords an *S* = 1 Fe(IV) peroxy-bridged intermediate of type **C1**, which decays through a triplet–quintet spin-crossing point to the ^5^[Fe^IV^ =O] species **D**.

#### Structure of the Fe(IV) oxido intermediate

A crucial factor controlling the chemoselectivity (i.e., halogenation *vs*. hydroxylation) and regioselectivity (i.e., which substrate C–H bond is activated) of NHFeHs is the relative position and orientation of the oxido oxygen of the ^5^[Fe^IV^ =O] intermediate with respect to the reactive C–H bond of the substrate. These are determined by the exact structure of the ^5^[Fe^IV^ =O] species and the position and conformation of the substrate.

For the Fe(IV) oxido complex, several coordination geometries are conceivable (Figure 7): If the succinate acts as a bidentate ligand, the complex will be six-coordinate octahedral (*OC*-6; **D1**, **D2**). If the succinate is monodentate, a range of trigonal-bipyramidal (*TBPY*-5; e.g., **D3**, **D4**) or square-pyramidal (*SPY*-5; e.g., **D5**, **D6**) structures are possible. The studies on the formation of **D** (see Section O2 activation and decarboxylation) almost invariably produced a *TBPY*-5 structure with the oxido ligand *trans* to N_235_ (**D3**). Kulik et al. (2009) obtained an *SPY*-5 isomer with apical N_116_ (**D6**), which they found to be slightly favored over the octahedral **D1** with bidentate succinate. **D6**, like **D3**, has the oxido *trans* to N_235_.

The most detailed investigation into the structure of ^5^[Fe^IV^ =O] in a NHFeH is the study by Solomon and co-workers (Wong et al., 2013), who used nuclear resonance vibrational spectroscopy (NRVS) in combination with BP86 (NRVS is essentially a kind of vibrationally resolved Mössbauer spectroscopy). By matching the experimental NRVS spectrum of the ^5^[Fe^IV^ =O] intermediate in SyrB2 with the non-natural substrate l-Cpg (l-cyclopropylglycine) to computed spectra of a range of structural models, they concluded that the correct structure is **D4**: A *TBPY*-5 isomer with axial oxido *trans* to N_116_. Their calculations of the O_2_ activation/decarboxylation pathway for both l-Cpg and natural l-Thr also yielded structure **D4**. However, for another non-natural substrate, l-Nva (l-norvaline = (2*S*)-2-aminopentanoate), isomer **D3** with oxido *trans* to N_235_ was obtained.

To account for the experimental Mössbauer spectrum of **D**, which indicates the presence of two ^5^[Fe^IV^ =O] species, Borowski et al. (2010) proposed an equilibrium between the *OC-6* isomers **D1** and **D2**, based on B3LYP calculations. However, the recent NRVS data appear to be incompatible with any six-coordinate isomer (Wong et al., 2013). To reconcile their NVRS-based proposal for isomer **D4** with the Mössbauer data, Wong et al. (2013) suggest that a variable number of hydrogen bonds to the oxido ligand is responsible for the observed Mössbauer spectrum.

The finding that the structure of **D** may depend on the substrate (**D4** for l-Thr and l-Cpg, but **D3** for l-Nva; Wong et al., 2013) highlights a very important aspect: the influence of the environment beyond the first coordination shell. The surrounding protein residues and the substrate bound in the active site can interact with the iron center and first-shell ligands, e.g., through hydrogen-bonding. In the specific case (Wong et al., 2013), the amino group of l-Nva formed a hydrogen bond to the proximal oxygen in the peroxy-bridged intermediate **C1**, effectively fixing this oxygen in a position *trans* to N_235_ and steering the reaction toward isomer **D3**, rather than **D4** as for the other substrates. The structure of the key intermediate **D** is therefore directly linked to the position and conformation of the substrate, which in turn depend on its interactions with surrounding residues.

### Hydrogen abstraction and radical rebound

The hydrogen abstraction and radical rebound steps **D** → **F** in NHFeHs have generally attracted the most computational interest as they are directly implicated in the question of chemoselectivity. However, the majority of studies employed small first-shell models, used an *a priori* assumed structure of **D** as a starting point (usually with oxido *trans* to N_235_, like **D3** or **D1**), and placed the substrate manually. These simplifications may in some cases limit the validity of the qualitative conclusions about the mechanism, as illustrated by the considerations in the previous section. However, we have attempted to include all available studies in the summary below.

The rate of decay of the [Fe^IV^ =O] species in SyrB2 has been measured experimentally for different substrates (Matthews et al., 2009a). Under the assumption that hydrogen abstraction is the only reaction channel, the free-energy barrier for hydrogen abstraction from l-Thr in SyrB2 is △^‡^G = 74 kJ mol^−1^, which may serve as a reference point in the following.

The first investigation of a NHFeH model (at the B3LYP level, using a first-shell model complex of type **D3** and propene as the substrate) reported the reaction profile for hydrogen abstraction and subsequent radical rebound of either hydroxyl or chlorine (de Visser and Latifi, 2009). The hydrogen-abstraction barrier from propene was △^‡^*H*_−*H*_(0 K) = 33 kJ mol^−1^. The rebound barriers were △^‡^*H*_+Cl_(0 K) = 44 kJ mol^−1^ and △^‡^*H*_+OH_(0 K) = 28 kJ mol^−1^, that is, in contradiction to the selectivity of the enzyme for chlorination. It was therefore postulated that the hydroxido complex **E** could react with the CO_2_ molecule liberated during the formation of **D** to form an intermediate with a chelating hydrogencarbonate, which was found to be a low-barrier reaction and slightly exothermic. Subsequent chlorine rebound, yielding 1-chloropropene, was calculated to be very facile and exothermic.

A preference for hydroxyl over chlorine transfer was also found in another study (at the B3LYP level, starting from hexacoordinate **D1)** (Pandian et al., 2009). With ethane as the substrate, △^‡^*G*_−H_ = 54 kJ mol^−1^, △^‡^*G*_+Cl_ = 30 kJ mol^−1^, and △^‡^*G*_+OH_ = 23 kJ mol^−1^. These authors considered if interactions with second-shell residues could restore the observed chemoselectivity. A mechanism was suggested whereby the hydroxido ligand in **E** is protonated through a nearby Arg–Glu pair, which would suppress hydroxylation. Chlorine rebound from the resulting aqua complex was indeed found to be very facile.

Kulik et al. (2009), having investigated the formation of **D** [see Section Formation of the Fe(IV) oxido intermediate], continued their PBE+*U* study for the rest of the reaction cycle. They found a coupled process with a low barrier of △^‡^*E*_−H/+Cl_ = 13 kJ mol^−1^, whereby **D6** abstracts the hydrogen from l-Thr, producing a hydroxido complex stabilized by a hydrogen bond from the OH ligand to Thr-OH, while the chlorine transfers to the forming substrate radical at the same time. Hydroxylation, on the other hand, requires a reorientation of the hydroxido ligand to break the hydrogen bond; the hydroxyl transfer itself has a barrier of ~17 kJ mol^−1^. The conclusion from that study is that chemoselectivity is controlled by the facile breaking of the Fe–Cl bond and the specific interactions between substrate and ^5^[Fe^IV^ =O] intermediate.

In a subsequent PBE+*U* study, Kulik and Drennan (2013) used a docking procedure (see Section Substrate placement in NHFeHs) to generate models of **D6** with different substrates: l-Thr, l-Nva, and l-Aba, which all react with SyrB2 but have different chemo- and regioselectivity patterns (Matthews et al., 2009b). They studied the dependence of hydrogen abstraction and rebound on the positioning of the substrate, specifically, on how deep the PPant arm reached into the active site. Their principal conclusions were: (i) The hydrogen abstraction barrier is rather insensitive to the “insertion depth” of the arm. The tether is conformationally sufficiently flexible to adjust such that the position of the reactive C–H remains practically unchanged over a broad range of insertion depths. (ii) The chemoselectivity, however, is controlled by the arm position: When the substrate is delivered “deeper” into the active site, it is preferentially chlorinated; when it is positioned farther out, hydroxylation is favored. The OH group of l-Thr, which is absent in l-Nva and l-Aba, was suggested to interact with second-sphere residues such that the substrate radical is positioned optimally for chlorine transfer, thus explaining the selectivity for chlorination of the natural substrate. The selectivity and reactivity patterns of l-Nva and l-Aba could similarly be rationalized based on their positioning in the active site.

A different explanation was provided by Borowski et al. (2010) based on structures, energies, and Mössbauer parameters calculated with B3LYP and validated by CASPT2 single-point energies. In their model, the substrate l-Thr was also positioned using a docking procedure (see Section Substrate placement in NHFeHs). They found that the isomeric *OC*-6 complexes **D1** and **D2** were almost equally stable and could interconvert (over a barrier of △^‡^*E* = 55 kJ mol^−1^), reproducing the experimental Mössbauer spectrum. Calculating the energetics of hydrogen abstraction and chlorine *vs*. hydroxyl rebound for both isomers, they concluded that the chemoselectivity for halogenation is determined by the slightly lower H-abstraction barrier from **D2** than from **D1** (△^‡^*E*_−H_ = 73 *vs*. 77 kJ mol^−1^). Once the respective hydroxido complex **E** is formed, the ligand *trans* to N_235_ (i.e., Cl for **D2**, OH for **D1**) preferentially and easily recombines with the substrate radical. The slight advantage of the pathway *via* **D2** was explained by favorable interactions of the oxido ligand with two water molecules included in the model.

Wong et al. (2013), having concluded that the correct [Fe^IV^ =O] isomer is **D4** (for the l-Thr substrate; see Section Structure of the Fe(IV) oxido intermediate), used B3LYP to calculate hydrogen abstraction energetics. They obtained a barrier of △^‡^*G*_−H_ = 100 kJ mol^−1^ and a slightly endergonic reaction energy of △_r_*G*_−H_ = 25 kJ mol^−1^. Importantly, in the resulting hydroxido complex derived from **D4**, the OH ligand is involved in hydrogen bonds with succinate and a water molecule and is also slightly further away from the substrate radical than the chlorine. OH rebound is therefore disfavored over Cl rebound, in agreement with experiment. Isomer **D3**, predicted to be formed with l-Nva substrate, is 22 kJ mol^−1^ less stable than **D4**. The barrier for hydrogen abstraction by **D3** is significantly lower (△^‡^*G*_−H_ = 70 kJ mol^−1^) than that for **D4**; the reaction is equally endergonic. In the corresponding hydroxido complex, the OH is not hydrogen-bonding and better positioned for rebound than the chlorine. This is again consistent with the experimental finding that SyrB2 hydroxylates l-Nva at a higher rate than it chlorinates l-Nva.

**Figure 8:**
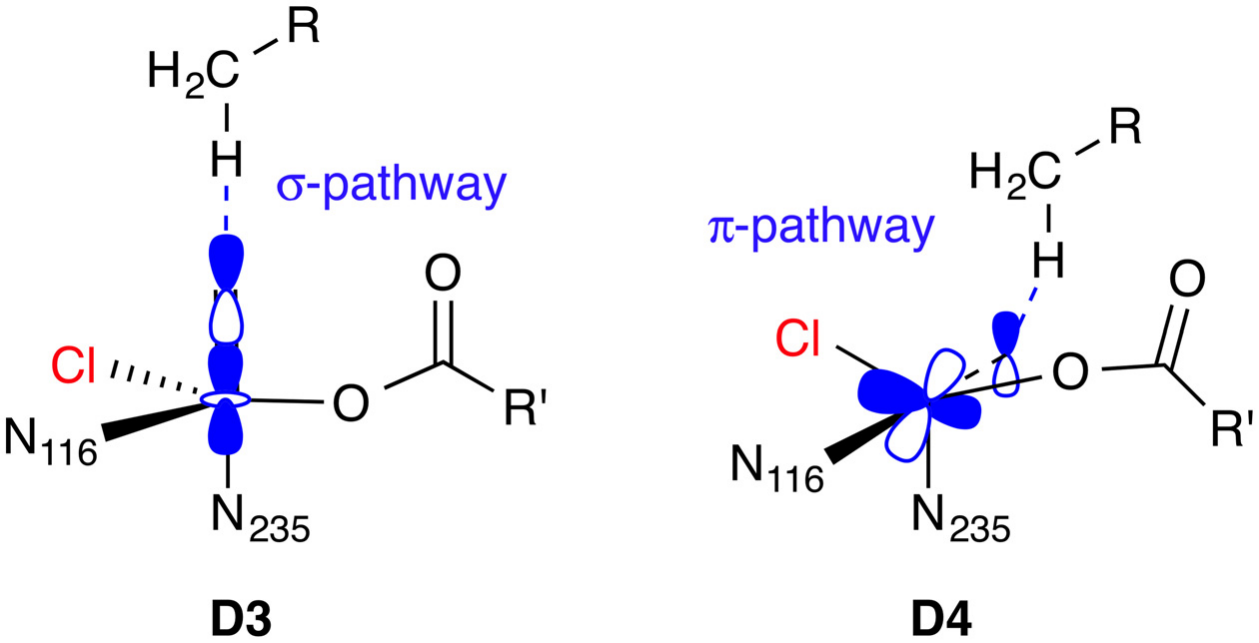
**Hydrogen abstraction pathways for ^5^[Fe^IV^ =O] isomers D3 and D4**. In the σ-pathway, an electron from the C–H bond is accepted into the σ^*^(Fe=O) orbital, hence an attack angle of 180° is ideal. In the π-pathway, the acceptor orbital is a π^*^(Fe=O), so the ideal attack angle is around 120°.

The difference in reactivity toward hydrogen abstraction between the [Fe^IV^ =O] isomers **D3** and **D4**, or more generally, between a linear *vs*. side-ways attack of the C–H bond on the Fe=O unit, has been studied in detail for various small [Fe^IV^ =O] complexes (Chen et al., 2010a; Geng et al., 2010; Janardanan et al., 2010; Ye and Neese, 2011) using B3LYP and CCSD(T) methods. In brief, the actual reactive form, which evolves as the Fe=O bond is slightly stretched as the transition state is approached, is best described as [Fe^III^−O^•^]. On the quintet surface, the spatial orientation of the reactive orbitals in ^5^[Fe^III^−O^•^] favor a linear attack (σ-pathway) over a side-ways attack (π-pathway); see Figure 8. The preference is reversed on the triplet surface. In the active site of an enzyme, the relative position and orientation of the reactive Fe=O and C–H bonds are restrained by the protein environment. The attack pathway is therefore controlled, or at least strongly influenced, by steric requirements, rather than intrinsic electronic preferences. Moreover, the coordination geometry and ligand environment of the iron center, which are also influenced by the protein, determine whether the most favorable acceptor orbital in any given spin state is of σ- or π-character. QM/MM studies on the NHFe/2-OG hydroxylase AlkB (Fang et al., 2013; Quesne et al., 2014), taking into account the full protein environment, indeed found attack pathways for C–H abstraction that deviated sometimes substantially from an ideal σ- or π-attack geometry.

## Discussion

### Chemistry of enzymatic halogenation

Three mechanistic classes of halogenating enzymes are currently known where at least one member has been structurally characterized: electrophilic (haem-, vanadate-, or flavin-dependent), nucleophilic (SAM-dependent), and radical (non-haem iron/2-oxoglutarate dependent) halogenases. These enzymes are required for the biosynthesis of the relatively small, but highly diverse group of natural organohalogens. From the chemical point of view, it is notable that oxidative pathways to activate the halide substrate, as employed by the electrophilic halogenases and NHFeHs, are the most prevalent (Vaillancourt et al., 2006). A plausible explanation for this is the multitude of oxidative processes in biosynthetic pathways in general and the associated ample collection of enzymes catalyzing them. It would therefore have taken only a few relatively minor evolutionary tweaks to redeploy, e.g., a flavin- or NHFe/2-OG-dependent hydroxylase as an oxidative halogenase, as evidenced by the similarities between respective mechanisms discussed in Sections Electrophilic halogenases and Radical halogenases.

The one exception from this general rule is fluorine. Its chemical properties make it special in several respects, and there are good reasons why natural organofluorines are scarce compared to other halometabolites, despite fluorine being the most abundant halogen in the Earth's crust: (i) Most of the fluorine is locked in sparingly soluble fluoride minerals (e.g., CaF_2_). (ii) When in solution, the fluoride anion is extremely well solvated; its very favorable hydration energy (Δ_hyd_G^*^ ≈ −437 kJ mol^−1^) must be offset to remove fluoride from the aqueous environment. (iii) Fluorine's high electronegativity (high ionization energy, very positive reduction potential) precludes its participation in the oxidative/electrophilic or radical halogenation pathways, which are common for the other halogens; “F^+^” or “F^•^” equivalents are not accessible under physiological conditions.

However, when (partially) desolvated, F^−^ (and, to a lesser extent, the other halides) is a strong nucleophile, providing a route to fluorination *via* nucleophilic substitution (see Section Nucleophilic halogenases). The nucleophilic halogenases may be regarded as prime examples of “catalysis by desolvation” (Lohman et al., 2013). The question of how exactly the energetic cost incurred by stripping a solvated halide ion from its solvation shell is offset to create a viable halide binding site has not received much attention beyond the identification of specific hydrogen-bonding interactions between bound halide and residues in the binding site. The suggestion (Healy, 2011) that halide binding stabilizes the protein's secondary structure, i.e., that there is a more delocalized contribution from the protein environment beyond the immediate, directed interactions, certainly appears worthy of further investigations.

### Challenges to modern electronic-structure approaches

The studies into the mechanism of NHFeHs (Section Radical halogenases) provide a perfect illustration of the many challenges mechanistic enzymology poses for modern computational methods. Despite, or perhaps because, of these, this class of enzymes has been, and continues to be, intensely investigated. The problems start with the difficulty to find an adequate quantum-chemical method able to treat the complex electronic structure of the central iron complex that is both reasonably accurate and affordable. While DFT is usually the method of choice for most organic systems and many “well-behaved” transition-metal centers, it is not robust enough to deal reliably with the delicate qualitative changes in the electronic structure of the iron complex between spin states and oxidation states. Even the pragmatic solution of selecting the “right” DFT method by validating it against theoretical or experimental benchmarks is fraught with difficulties in the case of NHFe systems. As described in Section O_2_ activation and decarboxylation, different DFT methods, seemingly validated against spectroscopic and structural data, still yield qualitatively differing descriptions of the steps involved in the formation of the [Fe^IV^ =O] intermediate.

Benchmarking against high-level computations is equally problematic, not least because even a minimal model of the iron complex that preserves the essential effects of the ligands on the electronic structure still contains 15–20 atoms. This pushes against the practicability limits of modern electronic-structure methods, despite the ever-continuing increase in computing power and the methodical and algorithmic advances, notably, the broad availability of highly efficient explicitly correlated “R12” or “F12” coupled-cluster methods (Ten-no and Noga, 2012). Even if an (F12-)CCSD(T) calculation with a decent-size basis set is just about feasible (for recent examples of applications of coupled-cluster methods to NHFe systems, see, e.g., Chen et al., 2010a, 2011; Geng et al., 2010; Ye et al., 2012), one needs to interpret the results with care (Harvey, 2011; Neese et al., 2011). The close-lying electronic states, for which first-row transition metal complexes are notorious, may cause the coupled-cluster calculation to converge to the wrong electronic state, depending on the initial reference wave function used (e.g., HF, CASSCF, or DFT), yielding a qualitatively incorrect description of the electronic structure. For these reasons, coupled-cluster results can hardly serve as benchmarks for these systems, even though they are considered the “gold standard” in many other areas.

In principle, the approach of choice for this type of problem should be a correlated multireference method, such as CASPT2, NEVPT2, MRCI, or MRCC. However, the prohibitive cost of these methods require one to make judicious compromises on the model size, basis set, and number of correlated electrons, often to an extent that the results can again hardly be considered to be of unassailable benchmark quality. Nevertheless, especially the somewhat more affordable CASPT2 or NEVPT2 methods have served, often in combination with other data, to back up DFT calculations on NHFe-type systems (see, e.g., Borowski et al., 2010; Vancoillie et al., 2010; Ye et al., 2012). A promising alternative could be DMRG (density matrix normalization group) approaches, which have been demonstrated (Boguslawski et al., 2012) to provide an accurate description of the electronic structure in Fe–NO model systems where CASSCF fails qualitatively.

It is worth noting that the electronic-structure challenges encountered in NHFeHs are essentially the same as those in other NHFe and P450-type systems, which all share the electronically problematic Fe=O unit. However, in NHFeHs (and to some extent NHFe enzymes in general), these electronic problems are greatly exacerbated by, and coupled to, a number of additional complications: (1) The iron centers in NHFe enzymes have a variable, conformationally flexible coordination sphere (in contrast to the rigid porphyrin ligand of P450-type systems). In NHFe/2-OG dependent enzymes, the ligand sphere even changes over the course of the catalytic cycle, as 2-OG is decarboxylated to succinate. Moreover, five-coordinate species, which are intrinsically structurally flexible, are easily accessible. The halide ligand in NHFeHs, compared to the ligating Glu or Asp residue of NHFe hydroxylases, is conformationally unrestrained, allowing for even more structural flexibility. (2) No experimental structure with bound substrate (or inhibitor or product) is available for NHFeHs. (3) The substrate is not just a small molecule, but tethered to a carrier protein.

Each of the above points adds another level of (structural and therefore also electronic) uncertainty and complexity that needs to be accounted for and contained as best as possible by modeling approaches. Because the first-shell coordination sphere is so variable in these systems, it is also very easily modulated by interactions with the protein environment.

### Importance of the protein environment

Quite apart from the complexities of electronic structure, which may be considered a special problem of metalloenzymes containing redox-active first-row transition metals, the NHFeHs studies (Section Radical halogenases) also highlighted the importance of a proper treatment of the environment around the immediately reactive moieties. The presence or absence of interactions (often hydrogen bonds) with protein residues, water molecules, or other moieties present in the active site may control the accessibility of particular forms of the reactants (e.g., isomers, conformers, electronic states) and thus qualitatively affect the mechanism. There is certainly a role for studies on small, first-shell models, which may provide valuable insights into the intrinsic reactivity of the system. However, one needs to exercise caution when drawing wider conclusions, e.g., about the selectivity of the reaction in the enzyme.

There are two main approaches to incorporate the effects of the environment. One may select a (small to moderate) number of surrounding residues and treat the entire cluster at the quantum-chemical level (Siegbahn and Himo, 2011). The positions are taken either from a crystal structure or a molecular-dynamics snapshot. They need to be partially constrained during structure optimizations to mimic the missing steric constraints of the full protein. The problem with cluster models is that one has to choose the “relevant” environment residues *a priori* while keeping the model to a manageable size.

The second option are combined QM/MM methods (Warshel and Levitt, 1976; Senn and Thiel, 2007a,b, 2009; Wallrapp and Guallar, 2011; Chung et al., 2012), where the environment outside the quantum-chemically treated reactive core is described by a classical force field. Depending on the implementation, the MM environment interacts with the QM core sterically (mechanical embedding) and/or electronically (electrostatic embedding). In the latter case, the QM region is polarized by the classical partial charges of the environment. This is often a quantitatively, or even qualitatively, crucial factor. While a simpler mechanical-embedding model may be adequate in some cases, it would be very difficult to identify these with certainty *a priori*; an instructive comparison of the effects of different levels of QM/MM on the calculated reaction profile of an NHFe enzyme has recently been provided by Lundberg and Borowski (2013). Full QM/MM models are arguably the state-of-the-art approach to treat reactions in enzymes. Their biggest disadvantage is perhaps their intrinsic complexity, which requires one to control both QM and MM models, their coupling, and the ramifications of dealing with a “large” system with many degrees of freedom.

Having discussed the crucial role of the environment and some approaches to account for it in calculations, one also should recall the importance of accurate experimental structures as a starting point for all enzymatic reaction modeling. The best computational treatment is no use if the environment of the reactive center (in terms of composition and structure) is not sufficiently well known. Again, NHFeHs provide an excellent case study: The uncertainty introduced by the lack of an experimental structure with bound substrate or product is arguably one of the main reasons for the variety of contradictory mechanistic proposals. Computational chemists may occasionally tend to focus too strongly on perfecting the (quantum-chemical) description of the model and devote less attention to the preparatory steps of building the model, which mainly involve classical “molecular modeling” techniques like docking and molecular dynamics.

## Conclusion

The halogenases are a group of enzymes that have only come to the fore over the last 10 years thanks to the discovery of a number of novel enzymes. Although it is clear that the currently known halogenases can only be the tip of the iceberg and many more halogenating enzymes remain to be characterized, they already represent a remarkable variety of mechanistically unique approaches used by nature to introduce halogens into organic substrates. Computational studies using a range of approaches have started to elucidate many details of the mechanisms of these enzymes, often in synergistic combination with experiment. However, many questions that are amenable to present computational methods have so far remained open, making the halogenases a worthwhile target for future investigations. The studies discussed in this Review have also illustrated some of the limitations of the current computational approaches, which makes the halogenases a challenging testing ground for new methods and computational techniques.

### Conflict of interest statement

The author declares that the research was conducted in the absence of any commercial or financial relationships that could be construed as a potential conflict of interest.
